# *N-*thiocarboxyanhydrides, amino acid-derived enzyme-activated H_2_S donors, enhance sperm mitochondrial activity in presence and absence of oxidative stress

**DOI:** 10.1186/s12917-023-03593-5

**Published:** 2023-02-16

**Authors:** Eliana Pintus, Abigail F. Chinn, Martin Kadlec, Francisco Alberto García-Vázquez, Pavel Novy, John B. Matson, José Luis Ros-Santaella

**Affiliations:** 1 Department of Veterinary Sciences, Faculty of Agrobiology, Food and Natural Resources, Czech University of Life Sciences Prague, 16500 Prague, Czech Republic; 2 Department of Chemistry, Virginia Tech Center for Drug Discovery, and Macromolecules Innovation Institute, Virginia Tech, Blacksburg, VA 24061 USA; 3 Departamento de Fisiología, Facultad de Veterinaria, Campus de Excelencia Internacional Mare Nostrum, Universidad de Murcia, 30100 Murcia, Spain; 4 Department of Food Science, Faculty of Agrobiology, Food and Natural Resources, Czech University of Life Sciences Prague, 16500 Prague, Czech Republic

**Keywords:** Bioenergetics, Gasotransmitters, H_2_S-releasing agents, Reproductive biotechnologies, Sperm lifespan

## Abstract

**Background:**

Hydrogen sulfide (H_2_S) donors are crucial tools not only for understanding the role of H_2_S in cellular function but also as promising therapeutic agents for oxidative stress-related diseases. This study aimed to explore the effect of amino acid-derived *N*-thiocarboxyanhydrides (NTAs), which release physiological H_2_S levels in the presence of carbonic anhydrase, on porcine sperm function during short-term incubation with and without induced oxidative stress. For this purpose, we employed two H_2_S-releasing NTAs with release half-lives (*t*_1/2_) in the range of hours that derived from the amino acids glycine (Gly-NTA) or leucine (Leu-NTA). Because carbonic anhydrase is crucial for H_2_S release from NTAs, we first measured the activity of this enzyme in the porcine ejaculate. Then, we tested the effect of Gly- and Leu-NTAs at 10 and 1 nM on sperm mitochondrial activity, plasma membrane integrity, acrosomal status, motility, motile subpopulations, and redox balance during short-term incubation at 38 °C with and without a reactive oxygen species (ROS)-generating system.

**Results:**

Our results show that carbonic anhydrase is found both in spermatozoa and seminal plasma, with activity notably higher in the latter. Both Gly- and Leu-NTAs did not exert any noxious effects, but they enhanced sperm mitochondrial activity in the presence and absence of oxidative stress. Moreover, NTAs (except for Leu-NTA 10 nM) tended to preserve the sperm redox balance against the injuries provoked by oxidative stress, which provide further support to the antioxidant effect of H_2_S on sperm function. Both compounds also increased progressive motility over short-term incubation, which may translate into prolonged sperm survival.

**Conclusions:**

The presence of carbonic anhydrase activity in mammalian spermatozoa makes NTAs promising molecules to investigate the role of H_2_S in sperm biology. For the first time, beneficial effects of NTAs on mitochondrial activity have been found in mammalian cells in the presence and absence of oxidative stress. NTAs are interesting compounds to investigate the role of H_2_S in sperm mitochondria-dependent events and to develop H_2_S-related therapeutic protocols against oxidative stress in assisted reproductive technologies.

**Supplementary Information:**

The online version contains supplementary material available at 10.1186/s12917-023-03593-5.

## Background

After being regarded as a mere poison for decades, hydrogen sulfide (H_2_S) is the third gaseous molecule or gasotransmitter^1^ that has been discovered to play a major role in many cellular physiological processes [1, 2]. In the male reproductive system, H_2_S is involved in spermatogenesis, sperm motility, and erectile function [3, 4]. Interestingly, men affected by fertility disorders show reduced seminal H_2_S levels and poor sperm motility, the latter being relieved after exogenous H_2_S supplementation [5]. In porcine spermatozoa, H_2_S supplementation protects sperm cells against the deleterious effects of oxidative stress [6], a result that seems to be enhanced when combined with nitric oxide (NO) donor [7]. As oxidative stress is frequently associated with male fertility disorders and reduced offspring survival [8, 9], exogenous H_2_S supplementation may have a potential therapeutic value in male infertility treatment.

H_2_S donors are not only promising therapeutic agents but also crucial tools in exploring and understanding the physiological role of H_2_S in cellular function [10–13]. Till recently, sulfide salt donors like NaHS and Na_2_S were the most employed H_2_S-releasing agents in biological studies. They are considered “clean” H_2_S donors since no by-product is released upon H_2_S formation. However, these compounds provoke a short and large release of H_2_S that is not representative of the physiological concentrations of this gasotransmitter in cells and tissues [14]. GYY4137 is another popular H_2_S donor, which releases H_2_S at a slow and more controlled rate (typically assumed to be on the order of days), but it also suffers from several drawbacks including the undefined mechanism of release and generation of by-products like carbon monoxide (CO), which is another gasotransmitter that acts in similar ways to H_2_S [13, 14]. For these reasons, in the past few years, a growing list of donors that mimic the endogenous H_2_S levels and release no or only benign by-products has been developed and is currently available for testing in biological studies. Their mechanism of release includes a variety of triggering stimuli like light, water, pH, biological thiols, reactive oxygen species (ROS), or enzymes [11].

**Fig. 1:**
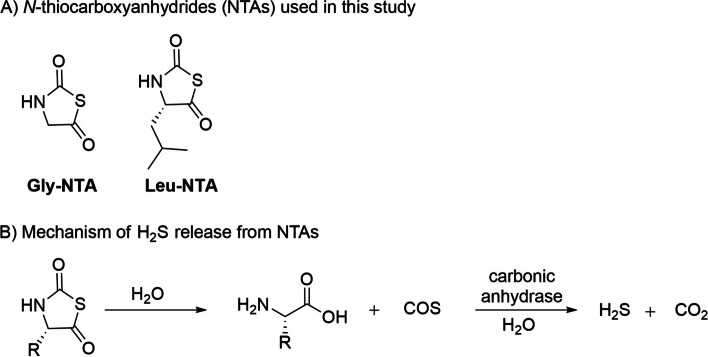
Chemical structure and mechanism of H_2_S release from *N*-thiocarboxyanhydrides (NTAs). Gly-NTA: NTA derived from glycine; Leu-NTA: NTA derived from leucine

Enzyme-triggered H_2_S donors are a novel group of H_2_S releasing agents with interesting but still little explored biological and therapeutic applications [13]. Several enzymes can be used as a trigger including, for instance, nitroreductase, esterase, glucose oxidase, and carbonic anhydrase. Among enzyme-triggered H_2_S donors, *N*-thiocarboxyanhydrides (NTAs) are carbonyl sulfide (COS)-mediated H_2_S donors, which release sustained and controlled H_2_S in the presence of the ubiquitous enzyme carbonic anhydrase, with innocuous peptide or amino acid by-products ([15–17] Fig. 1). Carbonic anhydrases are a family of widespread metalloenzymes responsible for the conversion of carbon dioxide (CO_2_) and water (H_2_O) to bicarbonate (HCO_3_^−^) and a proton (H^+^). Although less efficient than the canonical CO_2_ metabolism, carbonic anhydrases are also responsible for COS conversion to H_2_S [18]. A library of NTAs has been developed with different H_2_S release half-lives that range from 1 to 20 h depending on the conjugated amino acid [17]. NTAs have been shown to stimulate endothelial cell proliferation [15] and preserve the viability of cardiomyocytes [16], but their effects on the mammalian gametes are still unknown.

This study aimed to investigate the effect of NTAs on sperm function under different in vitro conditions: during short-term incubation and under induced oxidative stress. For these experiments, porcine spermatozoa were used as a cell model because of the known role of H_2_S in the sperm biology of this species [6, 7, 19, 20]. Because carbonic anhydrase function is essential for H_2_S release from NTAs, we first quantified the activity of this enzyme in each ejaculate component: sperm cells and seminal plasma. Then, we tested the effect of NTAs derived from glycine (Gly-NTA) or leucine (Leu-NTA) on sperm mitochondrial activity, plasma membrane integrity, acrosomal status, motility, kinetics, motile subpopulations, and redox balance during 3.5 h incubation at 38 °C and using hydrogen peroxide (H_2_O_2_) supplementation as a ROS-generating system.

## Results

### Experiment I. Measurement of carbonic anhydrase activity in porcine seminal plasma and spermatozoa

We found that 200 × 10^6^ spermatozoa showed carbonic anhydrase activity equal to 14.7 ± 5.2 nM/min/mL (*N* = 4). Notably, high carbonic anhydrase activity was found in the seminal plasma, being equal to 382.1 ± 88.4 nM/min/mL (*N* = 3). There was a large variability among replicates in enzymatic activity ranging from 9.9 to 22.1 nM/min/mL in the sperm cells and from 292.0 to 468.6 nM/min/mL in the seminal plasma. Although the ejaculates were supplemented with 5 mL Beltsville Thawing Solution (BTS; ~ 25% of ejaculate dose, see methods for further information) before seminal plasma extraction, the latter showed a considerably higher carbonic anhydrase activity compared to that of the sperm cells (*p* = 0.019, paired sample *t*-test, *N* = 3).

### Experiment II. Effect of NTAs on sperm function

#### Experiment IIa. Effect of NTAs on sperm parameters during 3.5 h incubation at 38 °C

**Fig. 2:**
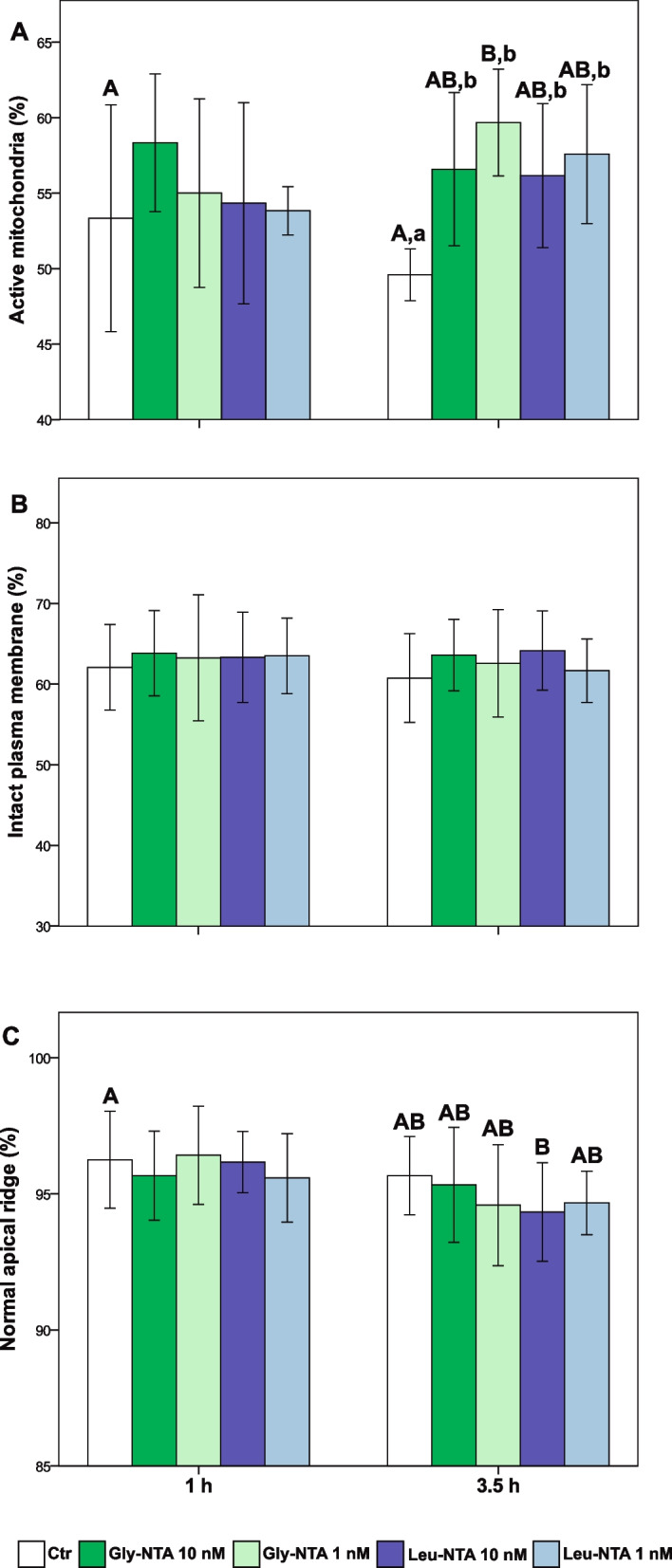
Effect of *N*-thiocarboxyanhydrides (NTAs) on porcine sperm function during 3.5 h incubation at 38 °C. **A** Mitochondrial activity; **B** Plasma membrane integrity; **C** Acrosomal status. Different small letters indicate statistically significant differences among treatments at each incubation time, while different capital letters indicate statistically significant differences among treatments at 3.5 h compared to the Control group at 1 h of incubation (*p* < 0.05). Ctr: control; Gly-NTA: NTA derived from glycine; Leu-NTA: NTA derived from leucine. Data are shown as mean ± SD of six replicates

At 1 h of incubation, there were no differences in sperm mitochondrial activity between the Control (Ctr) group and NTA treatments, although higher value of this parameter was observed in Gly-NTA 10 nM (*p* = 0.057, Fig. 2). At 3.5 h of incubation, all NTA treatments showed a significantly higher percentage of spermatozoa with active mitochondria compared to the Ctr group (*p* < 0.05), with the highest value observed in samples supplemented with Gly-NTA 1 nM (*p* < 0.001). This treatment also showed a significantly higher value of sperm mitochondrial activity at 3.5 h compared to that of the Ctr group at 1 h (*p* = 0.016), while the latter did not show any significant change in this parameter during incubation (*p* = 0.154). The NTAs did not show any significant effect on the percentages of spermatozoa with intact plasma membrane and acrosome compared to the Ctr group at each time of incubation (*p* > 0.05, Fig. 2). However, compared to the Ctr group at 1 h, Leu-NTA 10 nM showed a lower percentage of spermatozoa with intact acrosome at 3.5 h of sperm incubation (*p* = 0.033).

Albeit not significant, both NTAs at 1 and 10 nM showed higher total and progressive motility than that of the Ctr group both at 1 h and at 3.5 h of incubation (*p* > 0.05, Table 1). No significant differences were found in sperm kinetics and motile subpopulations between the Ctr group and NTA treatment groups at each incubation time (*p* > 0.05, Table 1, Supplementary Tables 1 and 2). Interestingly, while the progressive motility of the Ctr group did not significantly change between incubation times (*p* = 0.160), all NTA treatments showed higher progressive motility at 3.5 h than that of the Ctr group at 1 h of incubation (e.g., *p* = 0.007 and *p* = 0.014 for Gly- and Leu-NTA 10 nM, respectively). In contrast, while sperm velocity variables (i.e., average path velocity, VAP; curvilinear velocity, VCL; and straight-line velocity, VSL) in the Ctr group did not change during incubation, the samples supplemented with Gly- and Leu-NTA at the highest concentration (i.e., 10 nM) showed significantly lower values at 3.5 h compared to that of the Ctr group at 1 h (*p* < 0.05). In addition, the amplitude of lateral head displacement (ALH) of sperm cells from the Ctr group showed a significant decrease over incubation (*p* = 0.045), while there were no differences in this parameter between Gly-NTA 1 nM at 3.5 h and Ctr group at 1 h of incubation (*p* = 0.074).

**Table 1:** Effect of *N*-thiocarboxyanhydrides (NTAs) on boar sperm motility and kinetics during 3.5 h incubation at 38 °C

Time		Conc. (nM)	Total motility (%)	Progressive motility (%)	VAP (μm/s)	VCL (μm/s)	VSL (μm/s)	ALH (μm)	BCF (Hz)
1 h	Control		64.94 ± 9.06	63.27 ± 6.92^b^	85.86 ± 19.29^a^	120.94 ± 32.29^a^	80.00 ± 16.07^a^	4.92 ± 1.13^a^	18.35 ± 1.62
	Gly-NTA	10	68.10 ± 5.54	67.58 ± 4.60	87.06 ± 17.64	124.97 ± 30.75	80.97 ± 15.43	5.08 ± 1.12	17.47 ± 0.98
		1	69.54 ± 6.59	67.56 ± 6.85	85.17 ± 14.78	122.93 ± 26.66	79.39 ± 12.40	5.00 ± 1.05	17.78 ± 1.30
	Leu-NTA	10	68.72 ± 6.72	66.04 ± 4.97	86.37 ± 18.73	123.58 ± 31.64	80.30 ± 15.79	4.98 ± 1.13	18.41 ± 1.79
		1	69.29 ± 4.58	66.78 ± 7.47	84.53 ± 17.67	122.47 ± 32.17	77.97 ± 15.35	4.91 ± 1.09	18.12 ± 1.37
3.5 h	Control		65.21 ± 10.34	67.98 ± 8.99^ab^	70.91 ± 12.84^ab^	95.41 ± 20.58^ab^	67.69 ± 11.36^ab^	3.90 ± 0.73^b^	19.57 ± 0.70
	Gly-NTA	10	69.62 ± 6.15	72.34 ± 5.48^a^	68.05 ± 18.20^b^	92.51 ± 26.50^b^	65.16 ± 17.19^b^	3.79 ± 1.02^b^	18.65 ± 1.84
		1	67.72 ± 5.77	70.35 ± 6.91^a^	72.04 ± 12.10^ab^	97.20 ± 21.18^ab^	69.26 ± 11.04^ab^	4.00 ± 0.68^ab^	19.16 ± 1.13
	Leu-NTA	10	67.95 ± 3.39	71.51 ± 3.49^a^	67.22 ± 13.51^b^	88.74 ± 20.96^b^	64.80 ± 12.69^b^	3.69 ± 0.72^b^	19.63 ± 0.69
		1	67.71 ± 3.48	69.87 ± 6.27^a^	69.96 ± 15.32^ab^	93.40 ± 23.98^ab^	67.21 ± 13.99^ab^	3.86 ± 0.87^b^	19.43 ± 0.45

No statistically significant differences were found among treatments at each incubation time. Different letters within the same column indicate statistically significant differences among treatments at 3.5 h compared to the Control group at 1 h of incubation (*p* < 0.05). *ALH* Amplitude of lateral head displacement, *BFC* Beat-cross frequency, *Conc.* Concentration, *Gly-NTA* Glycine conjugated with NTA, *Leu-NTA* Leucine conjugated with NTA, *VAP* Average path velocity, *VCL* Curvilinear velocity, *VSL* Straight-line velocity. Data are shown as mean ± SD of six replicates

#### Experiment IIb. Effect of NTAs on sperm parameters under H_2_O_2_-induced oxidative stress

**Fig. 3:**
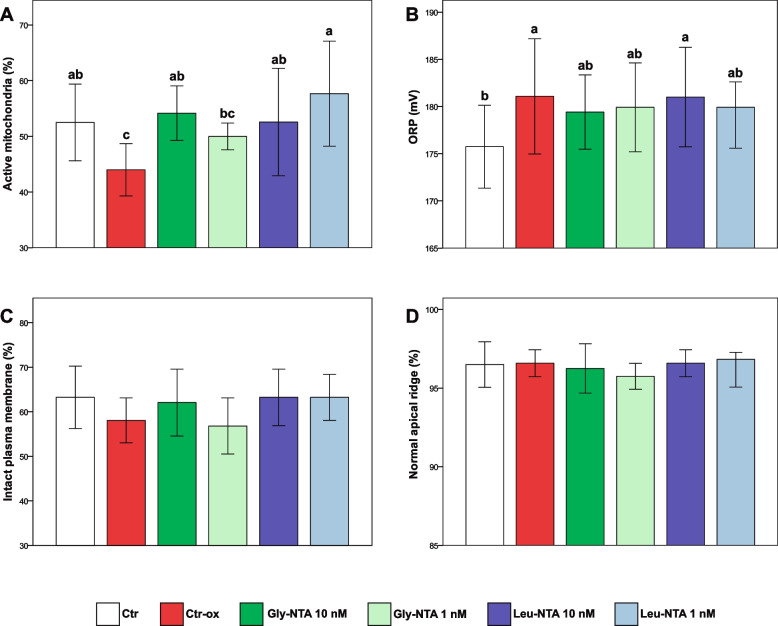
Effect of *N*-thiocarboxyanhydrides (NTAs) on porcine sperm function under H_2_O_2_-induced oxidative stress. **A** Mitochondrial activity; **B** Redox status; **C** Plasma membrane integrity; **D** Acrosomal status. Samples were analyzed after 1.5 h incubation at 38 °C. Oxidative stress was induced by exposing sperm cells to H_2_O_2_ 10 μM. Different letters indicate statistically significant differences among treatments (*p* < 0.05). Ctr: control; Ctr-ox: control under induced oxidative stress. Gly-NTA: NTA derived from glycine; Leu-NTA: NTA derived from leucine; ORP: oxidation–reduction potential. Data are shown as mean ± SD of six replicates

Oxidative stress induced by 10 μM H_2_O_2_ provoked a significant increase in the seminal oxidationreduction potential (ORP) and a decrease in sperm mitochondrial activity, total motility, kinetics, and the percentage of rapid and progressive spermatozoa (*p* < 0.05, Fig. 3, Table 2, Supplementary Tables 3 and 4). There were no significant differences between Ctr groups with and without oxidative stress in the sperm plasma membrane and acrosome integrity (*p* > 0.05, Fig. 3).

**Table 2:** Effect of *N*-thiocarboxyanhydrides (NTAs) on boar sperm motility and kinetics under H_2_O_2_-induced oxidative stress

	Conc. (nM)	Total motility (%)	Progressive motility (%)	VAP (μm/s)	VCL (μm/s)	VSL (μm/s)	ALH (μm)	BCF (Hz)
Control		78.07 ± 4.35^a^	74.61 ± 3.63^a^	89.21 ± 6.92^a^	122.21 ± 16.38^a^	84.01 ± 5.86^a^	4.89 ± 0.51^a^	18.98 ± 0.73^a^
Control-ox		58.63 ± 6.75^b^	68.47 ± 7.02^ab^	42.78 ± 12.60^b^	67.22 ± 15.91^b^	41.25 ± 12.30^b^	2.68 ± 0.69^b^	15.62 ± 0.82^b^
Gly-NTA	10	63.54 ± 7.61^b^	71.00 ± 4.92^ab^	43.23 ± 13.34^b^	68.04 ± 17.51^b^	41.64 ± 13.12^b^	2.71 ± 0.80^b^	15.49 ± 1.32^b^
	1	58.25 ± 12.93^b^	65.78 ± 11.54^b^	44.72 ± 13.99^b^	70.00 ± 18.21^b^	43.05 ± 13.69^b^	2.79 ± 0.81^b^	15.29 ± 1.55^b^
Leu-NTA	10	60.69 ± 8.01^b^	69.39 ± 4.88^ab^	39.98 ± 12.66^b^	62.18 ± 15.64^b^	38.46 ± 12.30^b^	2.48 ± 0.68^b^	15.77 ± 1.41^b^
	1	62.10 ± 7.83^b^	69.26 ± 5.08^ab^	40.18 ± 11.45^b^	62.96 ± 14.56^b^	38.77 ± 11.36^b^	2.53 ± 0.64^b^	15.52 ± 1.07^b^

Samples were analyzed after 1.5 h incubation at 38 °C. Oxidative stress was induced by exposing sperm cells to H_2_O_2_ 10 μM. Different letters within the same column indicate statistically significant differences among treatments (*p* < 0.05). *ALH* Amplitude of lateral head displacement, *BFC* Beat-cross frequency, *Conc.* Concentration, *Gly-NTA* Glycine conjugated with NTA, *Leu-NTA* Leucine conjugated with NTA, *Ox* oxidative stress, *VAP* Average path velocity, *VCL* Curvilinear velocity, *VSL* Straight-line velocity. Data are shown as mean ± SD of six replicates

The samples supplemented with Gly-NTA 10 nM and Leu-NTA 10 nM and 1 nM showed higher mitochondrial activity compared to the Ctr-ox group (*p* < 0.05, Fig. 3) and did not differ from the Ctr group without oxidative stress (*p* > 0.05). Among treatments, Leu-NTA 1 nM showed the highest sperm mitochondrial activity compared to the Ctr-ox group (*p* < 0.001). Moreover, the ORP of samples supplemented with Leu-NTA 1 nM and Gly-NTA 10 and 1 nM did not differ from the Ctr groups with and without induced oxidative stress (*p* > 0.05, Fig. 3). By contrast, Leu-NTA 10 nM and Ctr-ox treatments showed higher ORP values than that of the Ctr group (*p* < 0.05, Fig. 3).

No significant differences were found between the Ctr-ox group and NTA treatments on sperm motility, kinetics, plasma membrane integrity, and acrosomal status at any concentration tested (*p* > 0.05, Table 2, Supplementary Table 4).

## Discussion

Our study confirms that the enzyme carbonic anhydrase is found in the porcine ejaculate, in agreement with previous studies [21, 22]. For the first time, we measured carbonic anhydrase activity in each ejaculate component: the spermatozoa and the seminal plasma. Our results show that carbonic anhydrase activity is remarkably higher in the seminal plasma than in the sperm cells. The isoforms mostly expressed in the porcine seminal plasma and spermatozoa are carbonic anhydrase VI and II, respectively [21, 22]. The expression of carbonic anhydrase has been detected also in murine and human spermatozoa [23, 24], which makes NTAs suitable H_2_S donors also in sperm cells from other mammalian species. However, it should be noted that, based on the initial sperm concentration, semen pools were diluted from 24 to 32 times to reach the final concentration of 20 × 10^6^ spermatozoa per mL. This factor together with a certain degree of variability among replicates in seminal carbonic anhydrase activity may have influenced the effect of NTAs on sperm function. Moreover, although COS released by NTAs is quickly converted into H_2_S by carbonic anhydrase, we cannot rule out the possibility that some effects were mediated by COS, given its emerging role in chemical biology [18] as, for instance, COS has been detected in human exhaled-breath [25] and porcine coronary artery [26].

An interesting finding of this study is that both Gly- and Leu-NTAs enhance the mitochondrial activity of porcine spermatozoa with and without a ROS generating system. In the absence of oxidative stress, the positive effects of both Gly- and Leu-NTAs on sperm mitochondrial function became more pronounced throughout incubation most likely because of their H_2_S release half-lives, which may last up to 4.5 h [17]. This is the first study showing the beneficial effects of NTAs on mitochondrial activity in mammalian cells. Using esterase-triggered COS-H_2_S donor 1, Steiger et al. [27] observed a positive effect on mitochondrial respiration at low concentrations (i.e.,1 μM) but negative at high concentrations, mimicking the bell-shaped dose–response effect of H_2_S on mitochondrial bioenergetics. Stimulatory and protective effects of H_2_S on mitochondrial function have been described under physiological and pathological conditions [28, 29]. In mammalian cells, physiological concentrations of H_2_S enhance mitochondrial function by two main mechanisms: i) by increasing the activity of ATP synthase (complex V) and lactate dehydrogenase via cysteine *S*-sulphydration, which in turn stimulate the mitochondrial electron transport; ii) by donating electrons to the respiratory chain via sulfide quinone oxidoreductase and cytochrome *c* oxidase (complex IV) [30]. The observed increase in mitochondrial activity, however, only translates into a slight increase in sperm motility and kinetics. There has been a lively debate about the pathway that spermatozoa use to produce the energy needed for sperm motility [31–33]. Although both mechanisms coexist in the mammalian spermatozoa, some species mainly rely on oxidative phosphorylation (e.g., horse), while others on glycolysis (e.g., rodents). In porcine spermatozoa, glycolysis has been regarded as the predominant energetic pathway used for sperm motility [34], although recent evidence shows that sperm cells from this species heavily rely on oxidative phosphorylation for the ATP production needed for flagellar movements [35]. However, mammalian sperm cells probably do not exploit a single energetic pathway but show a certain degree of metabolic plasticity due to the diverse environments the spermatozoa encounter in their journey to reach the oocyte. Interestingly, while sperm progressive motility of the Ctr group did not significantly change over 3.5 h incubation, all treatments showed significantly higher progressive motility at 3.5 h incubation compared to that of the Ctr group at 1 h, which support the stimulatory effect of H_2_S on cell bioenergetics. It remains, nevertheless, to explore whether H_2_S released from NTAs may increase sperm survival during long-term storage, which can be useful for the improvement of semen preservation protocols. However, other than energy production, sperm mitochondria are also involved in sperm fertilization events like hyperactivation, calcium homeostasis, and ROS production [36]. Likewise, sperm mitochondrial dysfunctions are frequently associated with poor sperm quality (impaired sperm motility, DNA integrity, acrosome reaction) and male infertility both in natural conception and in vitro fertilization [37]. Under this perspective, NTAs represent not only an interesting tool to explore the implications of H_2_S during the fertilization process but they also may offer a therapeutic application for the improvement of assisted reproductive techniques.

Our findings show that the ROS generating system provoked by H_2_O_2_ supplementation increases the seminal ORP level, while it decreases sperm mitochondrial activity, motility, kinetics, and the percentage of sperm cells with fast and progressive trajectories. In contrast, other studies found that H_2_O_2_ > 250 μM had opposing effects on porcine sperm motility and mitochondrial activity during short-term incubation at 37 °C [38, 39]. This discrepancy might be due to individual variability across the animal population and methodological differences. For instance, the supplementation of semen extenders with antioxidants (e.g., bovine serum albumin, [40]) can potentially mask the reducing properties of the tested molecule. For this reason, in this study, we employed a hand-made diluent that does not contain any antioxidant that may influence the NTAs' effects. In line with a previous study [41], our findings confirm that oxidative stress is associated with high ORP levels in sperm samples. To the best of our knowledge, this is the first time that the measurement of ORP has been employed to check for the redox status in porcine spermatozoa. A growing number of recent studies indicate that ORP is a valuable tool for the evaluation of sperm redox balance and male fertility in humans (e.g., [42–44]). For instance, high ORP levels have been found in infertile men and are associated with low sperm number, motility, and normal morphology [43]. Unlike other oxidative stress analyses that only consider ROS or antioxidant levels, the ORP offers the advantage of a real-time and simultaneous evaluation of overall oxidants and antioxidants, thereby providing a more comprehensive and less time-consuming measurement of the sperm redox balance [45]. Both ROS levels and antioxidant defenses should be indeed considered to determine whether an insult can induce oxidative stress [9]. Using this approach, we found that under induced oxidative stress NTAs (except for Leu-NTA at the highest concentration) tend to reduce the seminal ORP, with the latter not different from that of the Ctr group without H_2_O_2_ supplementation. Taken together, our findings show that NTAs tend to preserve the sperm redox balance against the injuries provoked by high ROS levels, which provide further support to the antioxidant and positive effect of H_2_S on sperm function against oxidative stress [6].

Overall, our results provide further support for the protective role of H_2_S on mitochondrial activity under oxidative stress, in line with previous studies in other cell types (neurons: [46]; endothelial cells: [47]; cardiomyocytes: [48]). In endothelial cells exposed to low and steady-state levels of H_2_O_2_, nanomolar concentrations of the mitochondrially-targeted H_2_S donor AP39 exert cytoprotective effects and stimulate mitochondrial respiration [47]. In contrast, we did not find any beneficial effect of Na_2_S and GYY4137 on mitochondrial activity of boar spermatozoa exposed to Fe^2+^/ascorbate as a ROS generating system [6]. Except for Gly-NTA 1 nM, NTA treatments show a significantly higher mitochondrial activity compared to the Ctr-ox group and did not differ from the Ctr group without H_2_O_2_ supplementation, which indicates that the deleterious effects of oxidative stress were cushioned. The cytoprotective properties of COS/H_2_S donors like NTAs might be linked to protein *S*-persulfidation also known as *S*-sulphydration [49], which is also mediated by polysulfides (H_2_S_n_; [50]). In cardiomyocytes exposed to oxidative stress, Wang et al. [48] found that H_2_S promotes mitochondrial protection by down-regulating the expression of mitochondrial NOX4, caspase-3, and Bax, while it inhibits the release of cytochrome *c* from mitochondria. In agreement with previous studies on other cell types [15–17], NTAs do not show any cytotoxic effect on sperm function at the concentration and time interval tested. Moreover, despite their different H_2_S release half-lives, there were no remarkable differences in the Gly- and Leu-NTA’s effects on sperm function.

## Conclusions

The presence of carbonic anhydrase activity in mammalian spermatozoa makes NTAs promising molecules to investigate the role of H_2_S in sperm biology. Our findings show that Gly- and Leu-NTAs enhance sperm mitochondrial activity in the presence and absence of a ROS generating system and provide further evidence that these amino acid-based H_2_S-releasing agents do not exert noxious effects on cellular function. Moreover, the NTAs tested here tend to preserve sperm redox balance under oxidative stress conditions. These enzyme-triggered H_2_S donors represent a valuable tool to investigate the role of H_2_S in sperm mitochondria-dependent events such as fertilization and to develop H_2_S-related therapeutic protocols against oxidative stress in assisted reproductive technologies.

## Methods

### Reagents

All reagents were purchased from Sigma-Aldrich (Prague, Czech Republic), unless otherwise specified. Gly-NTA (C_3_SO_2_NH_3_, 117 g/mol) and Leu-NTA (C_7_NSO_2_H_11_, 173 g/mol) were synthesized in the laboratory as previously described [17]. In presence of 300 nM carbonic anhydrase, the Gly-NTA and Leu-NTA H_2_S release half-lives are 1.7 h and 4.5 h, respectively [17]. NTA chemical structure and mechanism of H_2_S release are shown in Fig. 1. WSP-5 was synthesized in the laboratory as previously described [51].

### Sample collection and preparation

Porcine sperm-rich ejaculate fractions (20 mL) were collected by the gloved-hand method from healthy and fertile Duroc boars at an animal breeding center (Lipra Pork, Czech Republic) and transported to the laboratory at 17 °C. At the laboratory, samples were immediately supplemented with 5 mL of BTS (in 1 L of doubled distilled H_2_O: D-glucose 37 g, sodium citrate 6 g, ethylenediaminetetraacetic acid 1.25 g, sodium bicarbonate 1.25 g, potassium chloride 0.75 g, gentamycin sulphate 250 mg; [52, 53]). The BTS’ pH (adjusted with NaOH 10 M) and osmolality were ~ 7.2 (Five Easy F20, Mettler-Toledo, Switzerland) and ~ 330 mOsm/kg H_2_O (Osmomat 3000, Gonotec, Germany), respectively. The use of hand-made diluent allows eliminating any potential influence of antioxidants or other compounds that might be found in commercial extenders. However, because of biosecurity measures of the breeding center, samples could not be diluted directly on site in our hand-made extender. This factor however did not likely affect our findings as all samples were treated under the same conditions and initial sperm motility was ≥ 75%. An aliquot of each ejaculate was collected and fixed in 0.3% formaldehyde in phosphate-buffered saline (PBS) solution for the evaluation of sperm abnormalities: only the ejaculates showing < 25% abnormal spermatozoa were used for the experiments. Evaluation (cell counting) of sperm morphology, mitochondrial activity, plasma membrane integrity, and acrosomal status were performed using the app-based cell counter designed by Thurman et al. [54].

### Experiment I. Measurement of carbonic anhydrase activity in porcine seminal plasma and spermatozoa

For the determination of carbonic anhydrase activity in the seminal plasma, a pool of two or three ejaculates was centrifuged at 14,000 g for 5 min at 4 °C. The supernatant was then transferred into another tube and centrifuged under the same conditions previously described. After that, the supernatant was collected and immediately stored at -80 °C. For the determination of carbonic anhydrase activity in spermatozoa, a pool of the same ejaculates that were used for the enzymatic assay in the seminal plasma was diluted into BTS at the final concentration of 200 × 10^6^ spermatozoa/mL. Samples were centrifuged at 14,000 g for 5 min at 4 °C; then, the supernatant was removed, and the sperm pellet was resuspended into an equal volume of cold PBS (4 °C). After another centrifugation (using the same conditions previously described), the supernatant was removed, and the pellet was resuspended into an equal volume of cold (4 °C) radioimmunoprecipitation assay (RIPA) buffer. Samples were then transferred into a stainless-steel micro vial, placed into an ultrasonic bath, and exposed to three cycles of sonication (45 Hz; 1 min sonication, 1 min cooling) in cold H_2_O. Sperm samples were then centrifuged at 14,000 g for 5 min at 4 °C. The supernatant was then collected and immediately stored at -80 °C. Seminal plasma and sperm samples were analyzed within 1 month after collection. Carbonic anhydrase activity was measured using a commercial assay kit (Sigma code: MAK404), following the manufacturer's instructions. The enzymatic activity was evaluated during 60 min of kinetic reaction. Because absorbance values of carbonic anhydrase activity in the seminal plasma were quickly above the limits of detection of the instrument, enzymatic activity was calculated over the first 10 min of reaction. A standard curve (r^2^ = 0.99) was prepared according to the kit instructions using nitrophenol solution (0–40 nM). All measurements were performed in duplicate at 405 nm at room temperature by a multimode microplate reader (Synergy H1, BioTek, CA, USA). Experiments were replicated four times using four semen pools (*N* = 10 boars).

### Experiment II. Effect of NTAs on sperm function

For each replicate, a pool of two or three boar ejaculates was centrifuged at 167 g for 3 min at 17 °C to remove debris and abnormal cells. The supernatant was then collected and checked for sperm concentration using a Bürker chamber (577.08 ± 56.22 × 10^6^ spermatozoa/mL, mean ± SD, *N* = 6). After that, samples were diluted to 20 × 10^6^ spermatozoa/mL using BTS. Sperm samples were randomly split into five (Experiment IIa) or six (Experiment IIb) groups. The Gly- and Leu-NTAs were prepared right before use by dissolving 1 mg of each NTA into a solution composed of 10% dimethylsulfoxide (DMSO) in H_2_O, at the final concentration of 1 mM. Serial dilutions were prepared to reach the final concentrations of 10 and 1 nM in sperm samples based on preliminary trials. The final DMSO concentration in all treatments was 0.0001%.

**Fig. 4:**
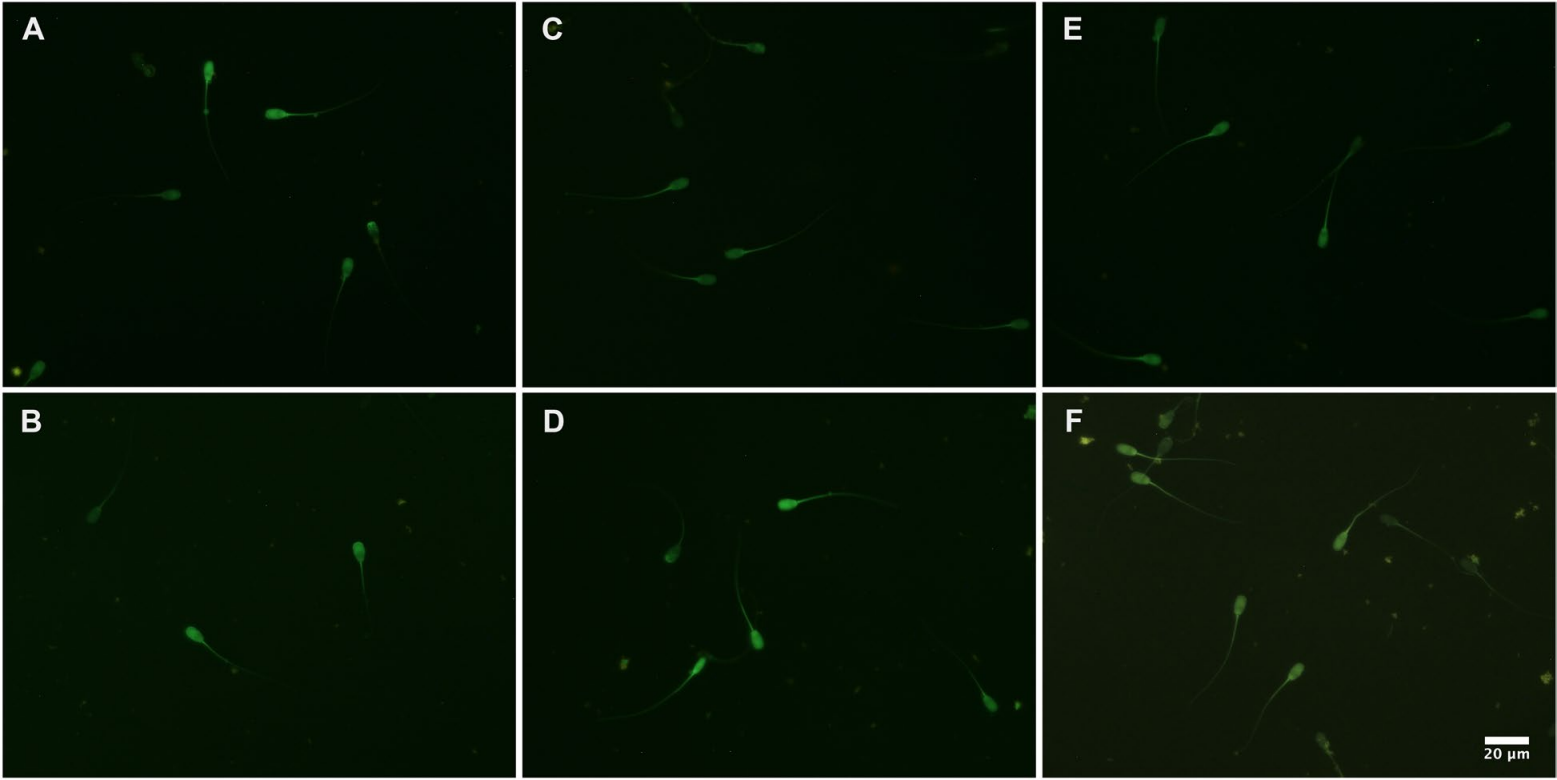
Representative images of hydrogen sulfide (H_2_S) release in porcine sperm cells supplemented with or without *N*-thiocarboxyanhydrides (NTAs) after 3.5 h of incubation at 38 °C. **A** Negative control group supplemented with dimethylsulfoxide only (vehicle); **B** Positive control group supplemented with the fast H_2_S-releasing donor Na_2_S 30 μM; **C** Samples supplemented with NTA derived from glycine (Gly-NTA) 10 nM; **D** Samples supplemented with Gly-NTA 1 nM; **E** Samples supplemented with NTA derived from leucine (Leu-NTA) 10 nM; **F** Samples supplemented with Leu-NTA 1 nM. Samples were stained with H_2_S selective fluorescent probe WSP-5

To confirm H_2_S release from NTAs, sperm samples were stained with H_2_S selective fluorescence probe WSP-5 using the protocols previously described [51], with minor modifications. Briefly, 20 μL of sperm samples (20 × 10^6^ spermatozoa/mL) were stained with a solution composed of 1 μL of WSP-5 (1 mg/250 μL in DMSO) and 79 μL of PBS. The samples were then incubated at 38 °C for 30 min in the dark. After that, samples were centrifuged twice at 500 g for 5 min at room temperature. Each time, the sperm pellet was resuspended in PBS. A sperm aliquot was then placed on a microscope slide and covered with a coverslip. Sperm cells were observed using epifluorescence microscopy (40 × objective; Nikon Eclipse E600, Nikon, Japan). One Ctr group was supplemented with DMSO (vehicle), while another Ctr group was supplemented with Na_2_S 30 μM (in PBS) freshly prepared before use. Representative pictures after 3.5 h of incubation at 38 °C are shown in the Fig. 4. Green fluorescence was observed in sperm cells from all treatments, including the Ctr group, which confirms the endogenous H_2_S production in porcine spermatozoa [20]. Fluorescence intensity could not be determined because of fast quenching. The fluorescence signal was localized both in the sperm head and flagellum.

### Experiment IIa. Effect of NTAs on sperm parameters during 3.5 h incubation at 38 °C

For the evaluation of NTAs on sperm function during short-term incubation, sperm samples were randomly split into 5 groups: Ctr, Gly-NTA 10 nM, Gly-NTA 1 nM, Leu-NTA 10 nM, and Leu-NTA 1 nM. The Ctr group was supplemented with DMSO (vehicle). Sperm mitochondrial activity, plasma membrane integrity, acrosomal status, motility, kinetics, and motile subpopulations were evaluated after 1 h and 3.5 h of sperm incubation at 38 °C in a water bath. The evaluations of sperm parameters after 1 h and 3.5 h of incubation were chosen based on the half-lives of Gly-NTA and Leu-NTA [17]. The experiment was performed the day after sample collection and replicated six times using six semen pools (*N* = 16 boars).

### Experiment IIb. Effect of NTAs on sperm parameters under H_2_O_2_-induced oxidative stress

For the evaluation of NTAs on sperm function under induced oxidative stress, sperm samples were randomly split into 6 groups: Ctr, Ctr under induced oxidative stress (Ctr-ox), Gly-NTA 10 nM, Gly-NTA 1 nM, Leu-NTA 10 nM, and Leu-NTA 1 nM. Oxidative stress was induced by supplementing all treatments (except Ctr group) with H_2_O_2_ 10 μM freshly prepared before use. Both Ctr groups were supplemented with DMSO (vehicle). Sperm mitochondrial activity, plasma membrane integrity, acrosomal status, motility, kinetics, motile subpopulations, and redox balance were evaluated after 1.5 h of sperm incubation in water bath at 38 °C. This time of incubation was chosen based on preliminary trials in which we observed a significant decrease of sperm motility after 1.5 h of H_2_O_2_ supplementation. The experiment was performed on the day of semen collection and replicated six times using six independent semen pools (*N* = 16 boars).

### Sperm mitochondrial activity

Mitochondrial status was evaluated as previously described [6]. Briefly, aliquots of sperm samples were incubated with rhodamine 123 (5 mg/mL in DMSO) and propidium iodide (0.5 mg/mL in PBS) for 15 min at 38 °C in the dark. At the end of sperm incubation, samples were centrifuged at 500 g for 5 min at room temperature. After removing the supernatant, the sperm pellet was resuspended in PBS. Then, 200 spermatozoa were evaluated by using epifluorescence microscopy (40 × objective): the spermatozoa showing bright green fluorescence in the midpiece were considered to have active mitochondria.

### Sperm plasmalemma integrity and acrosomal status

The sperm plasma membrane integrity and acrosomal status were evaluated as previously described [6]. Sperm plasma membrane integrity was evaluated after incubating sperm aliquots with carboxyfluorescein diacetate (0.46 mg/mL in DMSO), propidium iodide (0.5 mg/mL in PBS), and formaldehyde solution (0.3%) for 10 min at 38 °C in the dark. Then, 200 spermatozoa per sample were evaluated by using epifluorescence microscopy (40 × objective). The spermatozoa showing green fluorescence over the entire head were considered to have intact plasma membrane. Acrosomal status was assessed after sample fixation in 2% glutaraldehyde solution in PBS and examination using phase-contrast microscopy (40 × objective). Two hundred spermatozoa were evaluated per sample and the sperm cells with normal apical ridge were considered to have intact acrosome.

### Sperm motility and kinetics

Sperm motility was evaluated by using a Computer Assisted Sperm Analyzer (CASA; NIS-Elements; Nikon, Japan, and Laboratory Imaging, Czech Republic), which consists of an Eclipse E600 tri-ocular phase contrast microscope (Nikon, Japan) equipped with a 10 × negative phase-contrast objective (Nikon, Japan), a warming stage set at 38 °C (Tokai Hit, Japan), and a DMK 23UM021 digital camera (The Imaging Source, Germany). The analysis was carried out 90 s after loading 2 μL of sperm sample into a pre-warmed (38 °C) Leja chamber (Leja products B.V.; The Netherlands; chamber depth: 20 μm). The settings parameters were as follows: frames per second, 60; minimum frames acquired, 31; number of fields analyzed, 6; average path velocity (VAP) ≥ 10 μm/s to classify a spermatozoon as motile, straightness (STR) ≥ 80% to classify a spermatozoon as progressive [6]. A minimum of 200 sperm cells were analyzed for each sample*.* All videos were visually checked by the same researcher to remove debris or erroneously crossed sperm tracks. Sperm motile subpopulations were determined on the whole sperm population by cluster analysis.

### Seminal oxidation–reduction potential (ORP)

At the end of sperm incubation, 1.7 mL of sperm sample from each treatment was centrifuged at 16,300 g for 10 min at room temperature. Then, 700 μL of the supernatant was transferred into a microcentrifuge tube and incubated at 38 °C for a minimum of three minutes before reading. The ORP was measured using a micro ORP electrode with a platinum ring connected to a pH meter (Five Easy F20, Mettler-Toledo, Switzerland). The ORP of each sample was recorded after embedding the microelectrode into the solution for 3 min. After each sample analysis, the probe was calibrated into a redox buffer solution (220 mV, pH 7, Mettler-Toledo, Switzerland) for 30 s. The assay was run in duplicate per sample and expressed in millivolts (mV). The ORP levels were not normalized [55], because the experiments were performed at the same sperm concentration (i.e., 20 × 10^6^/mL). The ORP was evaluated only in samples submitted to induced oxidative stress (i.e., experiment IIb).

### Statistical analysis

Data were analyzed with the statistical program SPSS, version 20 (IBM Inc., Chicago, IL, USA). The Shapiro–Wilk’s and Levene’s tests were used to check the normal distribution and the equality of variances of the data, respectively. The paired-sample *t*-test was applied to check for differences in carbonic anhydrase activity between sperm cells and seminal plasma. The generalized linear model (GZLM) was used to analyze the effects of time and treatment on the sperm variables. To determine sperm motile subpopulations, a two-step cluster analysis was applied on the whole sperm population using sperm average path velocity (i.e., VAP) and trajectory straightness (i.e., STR) as variables. The number of clusters was automatically determined using the Euclidean distance measure and the Schwarz’s Bayesian criterion. After that, the number of clusters previously obtained was used to set up the K-Means cluster analysis by using the iteration and classification method. Independent sample *t*-test was used to check for differences between sperm motile subpopulations in kinetic variables. Data are shown as the mean ± SD. The statistical significance was set at *p* < 0.05.

## Supplementary Information

**Additional file 1:** **Supplementary Table 1.** Boar sperm motile subpopulations during 3.5 h incubation at 38 °C. **Supplementary Table 2.** Effect of *N*-thiocarboxyanhydrides (NTAs) on boar sperm motile subpopulations during 3.5 h incubation at 38 °C. **Supplementary Table 3.** Boar sperm motile subpopulations under H_2_O_2_-induced oxidative stress. **Supplementary Table 4.** Effect of *N*-thiocarboxyanhydrides (NTAs) on boar sperm motile subpopulations under H_2_O_2_-induced oxidative stress.

**Additional file 2:** Dataset Experiment I.

**Additional file 3:** Dataset Experiment II (except sperm motile subpopulations).

**Additional file 4:** Dataset Experiment IIa Sperm motile subpopulations.

**Additional file 5:** Dataset Experiment IIb Sperm motile subpopulations.

## Data Availability

All data generated during this study are included in this article and its supplementary information files.

## Abbreviations

ALH: Amplitude of lateral head displacement
ATP: Adenosine triphosphate
Bax: Bcl-2-associated X protein
BFC: Beat-cross frequency
BTS: Beltsville Thawing Solution
CASA: Computer Assisted Sperm Analyzer
Conc.: Concentration
Ctr: Control
DMSO: Dimethylsulfoxide
DNA: Deoxyribonucleic acid
Gly-NTA: NTA derived from glycine
GZLM: Generalized linear model
Leu-NTA: NTA derived from leucine
NOX4: NADPH oxidase
NTA: *N*-thiocarboxyanhydride
ORP: Oxidation–reduction potential
PBS: Phosphate-buffered saline
RIPA: Radioimmunoprecipitation assay
ROS: Reactive oxygen species
SD: Standard deviation
VAP: Average path velocity
VCL: Curvilinear velocity
VSL: Straight-line velocity

## Footnotes

1 Although commonly referred to as gaseous molecules, H_2_S besides nitric oxide (NO) and carbon monoxide (CO) are solutes rather than gases when they act as signaling agents [18].
