# Correction to: *N-*thiocarboxyanhydrides, amino acid-derived enzyme-activated H_2_S donors, enhance sperm mitochondrial activity in presence and absence of oxidative stress

**DOI:** 10.1186/s12917-023-03624-1

**Published:** 2023-05-10

**Authors:** Eliana Pintus, Abigail F. Chinn, Martin Kadlec, Francisco Alberto García‑Vázquez, Pavel Novy, John B. Matson, José Luis Ros‑Santaella

**Affiliations:** 1 Department of Veterinary Sciences, Faculty of Agrobiology, Food and Natural Resources, Czech University of Life Sciences Prague, Prague, 16500 Czech Republic; 2 Department of Chemistry, Virginia Tech Center for Drug Discovery, and Macromolecules Innovation Institute, Virginia Tech, Blacksburg, VA 24061 USA; 3 Departamento de Fisiología, Facultad de Veterinaria, Campus de Excelencia Internacional Mare Nostrum, Universidad de Murcia, Murcia, 30100 Spain; 4 Department of Food Science, Faculty of Agrobiology, Food and Natural Resources, Czech University of Life Sciences Prague, Prague, 16500 Czech Republic

**Correction: BMC Vet Res 19, 52 (2023).**

10.1186/s12917-023-03593-5

Following publication of the original article [1], the authors reported that a sentence was incomplete.

“Moreover, despite their diferent H” should be “Moreover, despite their different H S release half-lives, there were no remarkable differences in the Gly- and Leu-NTA’s effects on sperm function.”

The original article has been corrected.
