# Supplementary material for: N-thiocarboxyanhydrides, amino acid-derived enzyme-activated H2S donors, enhance sperm mitochondrial activity in presence and absence of oxidative stress
Source: BMC Vet Res. 2023 Feb 16;19:52. doi: 10.1186/s12917-023-03593-5 (PMC9933379; doi:10.1186/s12917-023-03593-5)
Supplement: Supplementary file 1 — Additional file 1: Supplementary Table 1. Boar sperm motile subpopulations during 3.5 h incubation at 38 °C. Supplementary Table 2. Effect of N-thiocarboxyanhydrides (NTAs) on boar sperm motile subpopulations during 3.5 h incubation at 38 °C. Supplementary Table 3. Boar sperm motile subpopulations under H2O2-induced oxidative stress. Supplementary Table 4. Effect of N-thiocarboxyanhydrides (NTAs) on boar sperm motile subpopulations under H2O2-induced oxidative stress. [file 12917_2023_3593_MOESM1_ESM.docx]

# Supplementary Tables

# Supplementary Table 1. Boar sperm motile subpopulations during 3.5 h incubation at 38 °C

|  |  | Number of cells | VAP (μm/s) | VCL (μm/s) | VSL (μm/s) | ALH (μm) | BCF (Hz) | STR (%) |
| --- | --- | --- | --- | --- | --- | --- | --- | --- |
| Rapid and progressive spermatozoa |  | 13,387 | 92.60±26.32^a^ | 129.22±45.60^a^ | 87.43±27.63^a^ | 5.24±1.96^a^ | 18.71±7.51^a^ | 94.35±11.01^a^ |
| Slow and no progressive spermatozoa |  | 11,955 | 11.34±14.46^b^ | 19.01±21.45^b^ | 9.82±13.68^b^ | 0.82±0.92^b^ | 11.51±7.18^b^ | 65.76±28.63^b^ |

Different letters within the same column indicate statistically significant differences between subpopulations (*p*<0.05). ALH: amplitude of lateral head displacement; BFC: beat-cross frequency; STR: straightness; VAP: average path velocity; VCL: curvilinear velocity; VSL: straight-line velocity. Data are shown as mean±SD of six replicates.

**Supplementary Table 2.** Effect of *N*-thiocarboxyanhydrides (NTAs) on boar sperm motile subpopulations during 3.5 h incubation at 38 °C

| Time |  | Conc. (nM) | Rapid and progressive spermatozoa (%) | Slow and no progressive spermatozoa (%) |
| --- | --- | --- | --- | --- |
| 1 h | Control |  | 56.01±15.24 | 43.99±15.24 |
|  | Gly-NTA | 10 | 58.20±12.39 | 41.80±12.39 |
|  |  | 1 | 58.90±10.71 | 41.10±10.71 |
|  | Leu-NTA | 10 | 58.98±13.82 | 41.02±13.82 |
|  |  | 1 | 58.19±11.25 | 41.81±11.25 |
| 3.5 h | Control |  | 46.55±13.85 | 53.45±13.85 |
|  | Gly-NTA | 10 | 49.32±11.63 | 50.68±11.63 |
|  |  | 1 | 49.37±10.52 | 50.63±10.52 |
|  | Leu-NTA | 10 | 45.31±12.34 | 54.69±12.34 |
|  |  | 1 | 47.06±11.73 | 52.94±11.73 |

There were no statistically significant differences among treatments (*p*<0.05). ALH: amplitude of lateral head displacement; BFC: beat-cross frequency; Conc.: concentration; Gly-NTA: glycine conjugated with NTA; Leu-NTA: leucine conjugated with NTA; VAP: average path velocity; VCL: curvilinear velocity; VSL: straight-line velocity. Data are shown as mean±SD of six replicates.

**Supplementary Table 3.** Boar sperm motile subpopulations under H_2_O_2_-induced oxidative stress

|  |  | Number of cells | VAP (μm/s) | VCL (μm/s) | VSL (μm/s) | ALH (μm) | BCF (Hz) | STR (%) |
| --- | --- | --- | --- | --- | --- | --- | --- | --- |
| Rapid and progressive spermatozoa |  | 7,310 | 67.32±26.15^a^ | 97.60±38.45^a^ | 64.62±25.56^a^ | 3.91±1.66^a^ | 17.16±7.48^a^ | 96.15±6.36^a^ |
| Slow and no progressive spermatozoa |  | 10,345 | 9.82±10.47^b^ | 20.51±18.55^b^ | 8.74±10.16^b^ | 0.82±0.83^b^ | 10.18±5.89^b^ | 70.77±26.94^b^ |

Samples were analyzed after 1.5 h incubation at 38 °C. Oxidative stress was induced by exposing sperm cells to H_2_O_2_ 10 μM. Different letters within the same column indicate statistically significant differences between subpopulations (*p*<0.05). ALH: amplitude of lateral head displacement; BFC: beat-cross frequency: STR: straightness; VAP: average path velocity; VCL: curvilinear velocity; VSL: straight-line velocity. Data are shown as mean±SD of six replicates.

**Supplementary Table 4.** Effect of *N*-thiocarboxyanhydrides (NTAs) on boar sperm motile subpopulations under H_2_O_2_-induced oxidative stress

|  | Conc. (nM) | Rapid and progressive spermatozoa (%) | Slow and no progressive spermatozoa (%) |
| --- | --- | --- | --- |
| Control |  | 74.80±4.80^a^ | 25.20±4.80^b^ |
| Control-ox |  | 35.19±15.38^b^ | 64.81±15.38^a^ |
| Gly-NTA | 10 | 38.57±20.08^b^ | 61.43±20.08^a^ |
|  | 1 | 37.55±20.48^b^ | 62.45±20.48^a^ |
| Leu-NTA | 10 | 33.82±19.45^b^ | 66.18±19.45^a^ |
|  | 1 | 35.10±18.91^b^ | 64.90±18.91^a^ |

Samples were analyzed after 1.5 h incubation at 38 °C. Oxidative stress was induced by exposing sperm cells to H_2_O_2_ 10 μM. Different letters within the same column indicate statistically significant differences among treatments (*p*<0.05). ALH: amplitude of lateral head displacement; BFC: beat-cross frequency; Conc.: concentration; Gly-NTA: glycine conjugated with NTA; Leu-NTA: leucine conjugated with NTA; Ox: oxidative stress; VAP: average path velocity; VCL: curvilinear velocity; VSL: straight-line velocity. Data are shown as mean±SD of six replicates.
